# Early intravenous immunoglobulin treatment in paraneoplastic neurological syndromes with onconeural antibodies

**DOI:** 10.1136/jnnp-2017-316904

**Published:** 2017-10-30

**Authors:** Giulia Berzero, Evgenia Karantoni, Caroline Dehais, François Ducray, Laure Thomas, Géraldine Picard, Véronique Rogemond, Gaëlle Candelier, Jean-Philippe Camdessanché, Jean-Christophe Antoine, Jérôme De Seze, Amélie Liou-Schischmanoff, Jérôme Honnorat, Jean-Yves Delattre, Dimitri Psimaras

**Affiliations:** 1 Service de Neurologie 2-Mazarin, AP-HP Groupe Hospitalier Pitié-Salpêtrière, Paris, France; 2 Neuroscience Consortium, University of Pavia, Monza Policlinico, and Pavia Mondino, Italy; 3 Centre de Référence National pour les Syndromes Neurologiques Paranéoplasiques, Hospices Civils de Lyon, Hôpital Neurologique Pierre Wertheimer, Lyon, France; 4 Service de Neurologie, Hôpital Nord, CHU Saint-Etienne, Saint-Etienne, France; 5 Université Claude Bernard Lyon 1, F-69372, Université de Lyon, Lyon, France; 6 Institut NeuroMyoGene, INSERMU1217/CNRS UMR 5310, Lyon, France; 7 Service de Neurologie, Hôpital Civil, Strasbourg, France; 8 Service Pharmacie, Pôle SPEPS, Hôpitaux Universitaires Pitié Salpetrière–Charles Foix, Paris, France; 9 Centre de Recherche de l’Institut du Cerveau et de la Moelle Epinière (CRICM), UMRS 975, Université Pierre et Marie Curie - Paris VI, Paris, France

**Keywords:** paraneoplastic neurological syndromes, onconeural antibodies, cancer treatment, immunotherapy, intravenous immunoglobulin

## Introduction

Paraneoplastic neurological syndromes (PNS) are immune-mediated complications of cancer, characterised by relentless progression. The mainstay of PNS treatment is the achievement of tumour remission,^1^ while immunotherapy provides only little additional benefit.^2 3^ However, in historical series, immunotherapy was initiated over 6 months after neurological onset and, at that stage, neuronal loss is already extensive and irreversible.

Among available immunotherapies, intravenous immunoglobulin (IVIg) has been used in single cases^4^ and in one retrospective series,^3^ showing some efficacy when administered timely.^4^ Based on these findings, we designed a prospective study to assess the efficacy and safety of early IVIg treatment in patients with PNS.

## Methods

### Study design

This prospective, multicentre, non-comparative, phase II clinical study was performed by the ‘Centre de Reference Français des Syndromes Neurologiques Paranéoplasiques’. Written informed consent was obtained from all participants. This trial is registered at ClinicalTrials.gov (NCT02343211).

### Participants

Inclusion criteria were: (1) diagnosis of ‘definite’ PNS^5^; (2) anti-Hu, anti-Yo, or anti-CV2/CRMP5 antibodies in the serum and/or in the cerebrospinal fluid; (3) neurological symptom onset within 6 months; (4) modified Rankin Score (mRS) 2 or 3; (5) neurological deterioration over the last 3 weeks. Exclusion criteria were: (1) other concomitant immunotherapy; (2) absolute contraindications to IVIg (hypersensitivity to IVIg, selective IgA deficiency); (3) thrombophilia; (4) renal insufficiency (creatinine clearance <30 mL/min).

### Interventions

Enrolled patients received three cycles of IVIg (Privigen, 2 g/kg, every 4 weeks), followed by an interim evaluation. If the patient was stable or improved according to the primary outcome measure, three additional IVIg cycles were administered. If the patient deteriorated, IVIg was discontinued. Final evaluation was performed at 6 months.

The primary endpoint was improvement on the mRS at 3 months (decrease of at least one point). Secondary endpoints were: improvement on the mRS at 6 months (decrease of at least one point), improvement on the International Cooperative Ataxia Rating Scale (ICARS) at 3 and 6 months in patients with cerebellar ataxia (decrease of at least 10 points) and improvement on the Overall Neuropathy Limitations Scale (ONLS) at 3 and 6 months in patients with peripheral neuropathy (decrease of at least one point).

Adverse events were classified according to the Common Terminology Criteria for Adverse Events (CTCAE) V.4.03.

In patients without a history of cancer, a search for an occult neoplasm was performed. Whenever indicated, tumour treatment was started promptly (according to the schedule established by the referring oncologist) and was performed in parallel with IVIg treatment.

### Post hoc analyses

Patients continued to be followed after the end of the 6-month study period, as part of the normal follow-up for their disease. Survival analyses were performed by the Kaplan-Meier method.

## Results

### Patient characteristics

The clinical features of the 17 patients are reported in table 1. Fourteen patients had anti-Hu, two patients had anti-CV2/CRMP5 and one patient had anti-Yo antibodies. Three patients had isolated central nervous system involvement, three patients had mixed central and peripheral impairment, while 11 patients had isolated peripheral neuropathies. In all patients, cerebrospinal fluid analysis showed inflammatory abnormalities. Thirteen patients had an associated cancer, 11 of whom received anti-tumour treatments in parallel with IVIg.

**Table 1:** Clinical characteristics of the 17 patients included in the present study

Point	Gender/age	Clinical presentation	Ab type	Delay PNS/IVIg (months)*	IVIg cycles (n)	Tumour histology	Delay PNS/tumour (months)*	Tumour treatment during IVIg treatment (±3 months)	Neurological outcome			Tumour status at 6 months	Last follow-up (months), patient status	Cause of death
									mRS at enrolment	mRS at3 months	mRS at 6 months			
1	F/85	SSN	Yo	2	6	Endometrium adenocarcinoma	42-	None	3	1	1	Complete response	40, Alive	NA
2	M/80	SSN	Hu	1.2	3	SCLC	1.7+	Cisplatin/VP16; carboplatin/VP16	3	5	5	Partial response	12, Alive	NA
3	F/63	LE	Hu	2	6	No tumour detected	NA	NA	2	1	1	No tumour detected	38, Alive	NA
4	F/62	SMN	Hu	4	3	Neuroendocrine breast cancer	23−	None	3	4	4	Stable disease	8, Alive	NA
5	M/58	MN	Hu	5.5	6	SCLC	6+	Carboplatin/VP16	3	3	3	Tumour progression	26, Dead	Tumour progression
6	M/53	SMN	Hu	3.8	1	SCLC	4+	Vinorelbine/cisplatin; cisplatin/VP16	3	NA	NA	NA	3.5, Dead	Tumour progression
7	M/67	SSN	Hu	2	6	SCLC	1.5+	Carboplatin/VP16	3	3	3	Stable disease	13, Dead	Tumour progression
8	M/76	SSN/ LEMS	Hu	3	6	SCLC	2.3+	Cisplatin/VP16; local RT; prophylactic brain RT	3	3	3	Stable disease	36, Alive	NA
9	M/60	PCD	CV2	3.5	6	No tumour detected	NA	NA	3	3	3	No tumour detected	32, Dead	Tumour progression (lung cancer)
10	M/56	SSN	Hu	3	6	SCLC	2+	Carboplatin/VP16; prophylactic brain RT	3	3	3	Stable disease	14, Dead	Tumour progression
11	M/58	SMN	Hu	4	0	No tumour detected	NA	NA	3	NA	NA	NA	4.5, Dead	Fall with head trauma
12	F/77	LE	Hu	2	2	SCLC	2+	Carboplatin/VP16; local RT	3	NA	NA	NA	3, Dead	Sepsis
13	M/48	SMN	Hu	1	2	SCLC	1+	Carboplatin/VP16	3	NA	NA	NA	2, Dead	PNS
14	M/39	SSN	Hu	3	4	Neuroendocrine rectum cancer	2+	Carboplatin/VP16; FOLFIRI; 5-FU/dacarbazine; local RT	2	2	NA	NA	6, Dead	Tumour progression
15	F/62	SSN/LEMS/ON	CV2	2	5	SCLC	2+	Carboplatin/VP16; local RT; prophylactic brain RT	2	2	3	Partial response	20, Alive	NA
16	F/57	SSN/LE	Hu	5	3	SCLC	6+	Cisplatin/VP16; local RT	3	3	4	Complete response	20, Alive	NA
17	M/59	LE/SSN	Hu	3.8	6	No tumour detected	NA	NA	3	3	3	No tumour detected	16, Alive	NA

*Delay from the onset of PNS to tumour detection: ‘+’ means the PNS precedes the tumour, while ‘−’ means the PNS follows the tumour.

Ab, antibody; F, female; FOLFIRI: FOLinic acid + Fluorouracil + IRInotecan; 5-FU: 5-fluorouracil; IVIg, intravenous immunoglobulin; LE, limbic encephalitis; LEMS, Lambert-Eaton myasthenic syndrome; M, male; MN, motor neuropathy; ON, optic neuropathy; PCD, paraneoplastic cerebellar degeneration; PNS, paraneoplastic neurological syndrome; Pt, patient; RT, radiotherapy; SCLC, small cell lung cancer; SMN, sensorimotor neuropathy; SSN, subacute sensory neuronopathy; VP-16: etoposide; mRS, modified Rankin Scale; NA, not applicable;.

### IVIg treatment

Median delay between neurological symptom onset and the start of IVIg treatment was 3 months (range 1–5.5). The median number of IVIg cycles per patient was 5.

### Neurological outcome

#### Primary endpoint

Of the 17 patients enrolled, 13 patients were evaluable at 3 months. The primary endpoint (improvement of the mRS at 3 months) was reached by two patients (12%) (patients 1 and 3). The expected threshold to consider the treatment effective (five patients) was not reached. Nine patients had a stable mRS (53%) and remained ambulatory, while two patients deteriorated on the mRS (12%) (patients 2 and 4).

#### Secondary endpoints

Twelve patients were evaluable at 6 months. At this time point, and compared with baseline mRS, two patients had improved (12%), six patients were stable (35%) and four patients had deteriorated (24%).

Scores on the neurological scales ONLS and ICARS were analysed. The ONLS showed improvement at 3 and 6 months in two patients (patients 7, 17) who were stable according to the mRS. The ONLS also showed deterioration at 6 months in one patient (patient 10) who was stable on the mRS. The ICARS was administered to a single patient with cerebellar degeneration (patient 9), showing a consistent improvement which, however, did not exceed the established threshold. Online supplementary figure 1 summarises the results from primary and secondary outcome measures.

10.1136/jnnp-2017-316904.supp1 Supplementary figure 1 Neurological outcome at3 (panel A) and at 6 (panel B) months. Histograms show the number of patients who improved, were stable or deteriorated on the modified Rankin Score (orange)and on specific neurological grading scales (Overall Neuropathy Limitation Scale or International Cooperative Ataxia Rating Scale) (purple) at the different time points. *Patients with isolated limbicencephalitis were evaluated with the modified Rankin Score, as no neurological grading scale for cognitive impairment was provided.

### Safety and tolerability

Four patients (24%) experienced grade 3 or 4 CTCAE: one patient had an allergic reaction (patient 6), one patient had a catheter infection (patient 10) and two patients developed sepsis (patients 12 and 15). Patient 12 died from sepsis. In the remaining three cases, the adverse event completely resolved with appropriate treatment.

### Mortality

Five patients died during the 6-month study period (patients 6, 11–14). Cause of death was tumour progression (two patients), PNS (one patient), sepsis (one patient) and fall with head trauma (one patient).

### Post hoc analyses

Patients were followed for a median follow-up of 13.7 months from enrolment (range 2.3–40.9). During the extension period, four additional patients died due to cancer progression (patients 5, 7, 9, 10). The median survival time in our cohort was 25.6 months.

## Discussion

This study is the first prospective trial that assesses the efficacy of IVIg treatment in patients with PNS. The goal of the study was to start immunotherapy as early as possible, at a stage where inflammation is prominent. This enrolment goal was achieved, as half of our patients were enrolled within 3 months of neurological symptom onset. Enrolment was restricted to ambulatory patients, as preserved ambulation was considered an encouraging feature. At 3 months from enrolment, most of our patients had improved (12%) or stabilised (53%) on the mRS, remaining ambulatory.

In order to be consistent with other PNS trials, we chose the mRS as the primary outcome measure. However, we observed that neurological grading scales captured minor improvements or deteriorations more accurately than the mRS. Future studies should consider using neurological grading scales as primary outcome measures.

Patients in whom a tumour was present received antitumour treatment in parallel with IVIg. Although tumour treatment could indeed have contributed to therapeutic results, neurological improvement was also detected in patients who did not receive concomitant tumour treatment (patients 1, 3, 9, 17), suggesting a beneficial independent effect of IVIg.

Four patients had a severe adverse event, which was ultimately fatal in one case (sepsis). Sepsis is a recognised cause for hospitalisation and death in cancer patients, and therefore it is impossible to distinguish the role of IVIg treatment in causing this complication.

In the present series, median overall survival time was 25.6 months, highlighting the recent dramatic increase in patient survival.^2 3^ Unlike in other reports,^2^ only one death in our study was directly attributable to the neurological disorder. These data support the view that immunotherapy should be administered as soon as possible, in order to stabilise the patient at an ambulatory status and prevent the life-threatening complications related to severe neurological disability.
