# Supplementary figures and images for: Early intravenous immunoglobulin treatment in paraneoplastic neurological syndromes with onconeural antibodies
Source: J Neurol Neurosurg Psychiatry. 2017 Oct 30;89(7):789–92. doi: 10.1136/jnnp-2017-316904 (PMC6031268; doi:10.1136/jnnp-2017-316904)

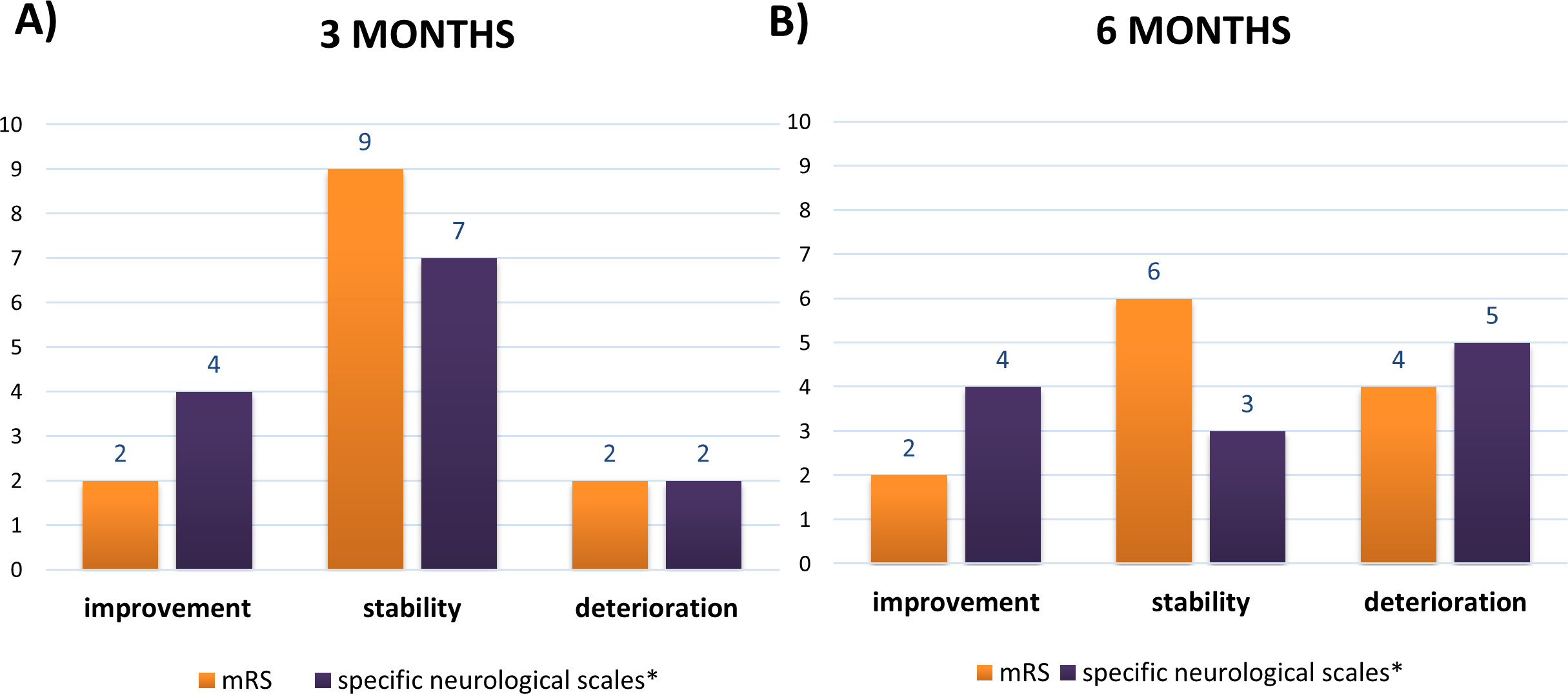

Supplement: Supplementary figure 1 [file jnnp-2017-316904supp001.jpg]
